# Cystatin C and long term risk of community-acquired sepsis: a population-based cohort study

**DOI:** 10.1186/s12882-015-0055-z

**Published:** 2015-04-23

**Authors:** Thomas Clark Powell, John P Donnelly, Orlando M Gutiérrez, Russell L Griffin, Monika M Safford, Henry E Wang

**Affiliations:** Department of Emergency Medicine, University of Alabama School of Medicine, 619 19th Street South, OHB 251, Birmingham, AL 35249 USA; Division of Nephrology, Department of Medicine, University of Alabama School of Medicine, Birmingham, Alabama USA; Department of Epidemiology, University of Alabama at Birmingham, Birmingham, Alabama USA; Division of Preventive Medicine, Department of Medicine, University of Alabama School of Medicine, Birmingham, Alabama USA

**Keywords:** Biomarkers, Cystatin C, Sepsis, Epidemiology

## Abstract

**Background:**

Chronic kidney disease (CKD) and systemic inflammation are risk factors for sepsis. While often viewed as a marker of chronic kidney disease, Cystatin C (Cyst-C) may also reflect systemic inflammation. We sought to determine the association between elevated baseline Cyst-C and long-term rates of community-acquired sepsis, and to determine if this relationship is influenced by traditional markers of CKD (estimated glomerular filtration rate [eGFR], albumin-to-creatinine ratio [ACR]) and inflammation (high sensitivity C-reactive protein [hsCRP]).

**Methods:**

We studied 30,239 adults ≥45 years old from the REasons for Geographic and Racial Differences in Stroke (REGARDS) cohort. The primary exposure was elevated Cyst-C (>1.12 mg/dL) measured at study baseline. The primary outcome was the first sepsis hospitalization during a 10-year observation period. Using Cox regression, we evaluated the association between elevated Cyst-C and first sepsis event, adjusted for sociodemographics, health behaviors, chronic medical conditions, eGFR, ACR and hsCRP.

**Results:**

Among participants, 1,532 experienced a sepsis event. Median Cyst-C levels were: sepsis 1.08 (IQR 0.91-1.33) mg/dL (43.8% >1.12 mg/dL), non-sepsis 0.94 (IQR 0.82-1.10) mg/dL (23.4% >1.12 mg/dL). Cyst-C > 1.12 mg/dL was independently associated with increased rates of sepsis, adjusted for participant demographics, health behaviors and chronic medical conditions (HR 1.75; 95% CI: 1.55-1.96). The addition of eGFR < 60 mg/min/1.73 m^2,^ ACR ≥ 30 mg/g and hsCRP > 3.0 mg/dL only partially attenuated the association between Cyst-C > 1.12 mg/dL and rates of sepsis (adjusted HR 1.51; 1.32-1.72).

**Conclusions:**

Elevated Cyst-C is associated with increased long-term rates of community-acquired sepsis, independent of abnormal eGFR, ACR or hsCRP. Cyst-C may play a role in long-term sepsis risk prediction and prevention.

## Background

Sepsis is the syndrome of microbial infection complicated by systemic inflammatory response. Sepsis is a major public health problem in the United States (US), encompassing 750,000 hospitalizations, 570,000 Emergency Department visits and 200,000 deaths annually [1-3]. The associated morbidity and mortality for sepsis is substantial, with 28-50% dying from related complications [4]. While numerous studies highlight the physiologic response to and care of acute sepsis, relatively little is known about risk factors that are associated with increased long-term risk of sepsis [5-11]. Identification of these risk factors could potentially lead to strategies for preventing or limiting the impact of sepsis in these individuals.

Cystatin C (Cyst-C) is a low molecular weight protein that is produced by all nucleated cells and removed from the bloodstream by glomerular filtration [12]. Cyst-C is most commonly recognized as a marker of glomerular function, with numerous studies evaluating its role in detecting chronic kidney disease (CKD), acute kidney injury and their associated sequelae [13-17]. However, studies of cardiovascular disease, systemic lupus erythematosus and malignancy highlight that Cyst-C may also act as a marker of more generalized inflammation [15,16,18-23]. CKD and inflammation are both associated with increased sepsis risk [9-11,24-27]. Despite these observations, few studies have evaluated the independent association of Cyst-C with future sepsis risk [28,29].

In this study, we sought to determine the association between Cyst-C measured at baseline and future risk of community-acquired sepsis.

## Methods

### Study design

We used data from the Reasons for Geographic and Racial Differences in Stroke (REGARDS) study, a national population-based longitudinal cohort. This study was approved by the Institutional Review Board of the University of Alabama at Birmingham. Written consent for participation was obtained by the parent REGARDS study.

### Data source

REGARDS is one of the largest ongoing national cohorts of community-dwelling adults in the US [30]. Created to examine risk factors for stroke, REGARDS contains 30,239 community-dwelling adults aged ≥45 years. The cohort is 45% male, 41% African American, and 69% >60 years old. The study oversampled black individuals and those living in the Southeastern US, with 21% of the cohort originating from the coastal plains of North Carolina, South Carolina and Georgia (the “stroke buckle”), and 35% from the remainder of North Carolina, South Carolina and Georgia plus Tennessee, Mississippi, Alabama, Louisiana and Arkansas (the “stroke belt”).

The REGARDS study recruited participants during 2003–2007. The study obtained baseline data for each participant using both phone interview and in-person evaluations. Baseline information included medical history, functional status, health behaviors, physical characteristics (height, weight), physiologic measures (blood pressure, pulse, electrocardiogram), and an inventory of medications. Additional data included diet, family history of diseases, psychosocial factors and prior residences. The study also collected blood and urine specimens from each participant.

Every 6 months, REGARDS contacted study participants by telephone to identify the date, location and attributed reason for all hospitalizations during the follow-up period. Study personnel then retrieved medical records for specific health events. If the participant died, the study team reviewed death certificates and medical records and interviewed proxies to ascertain the circumstances of the death.

### Outcomes - Identification of Sepsis Events

The primary outcome was a participant’s first hospitalization for a sepsis event. Using a strategy developed by Angus et al., we identified hospital admissions and emergency department visits attributed to a serious infection [3]. Trained abstractors reviewed relevant medical records to confirm the presence of infection on initial hospital presentation and its relevance as a major reason for hospitalization. Discordances were adjudicated among abstractors, with additional physician review as needed.

Consistent with international consensus definitions, we defined sepsis events as hospitalization for infection plus two or more systemic inflammatory response syndrome (SIRS) criteria. These include heart rate >90 beats/minute, fever (temperature >38.3°C or <36°C), tachypnea (>20 breaths/min) or carbon dioxide partial pressure (PCO2) <32 mmHg, and leukocytosis (white blood cells >12,000 or <4,000 cells/mm3 or >10% band forms) [2]. We determined vital signs and laboratory test results for the initial 28-hours of hospitalization. Because of our focus on “community-acquired” (vs. “hospital-acquired”) sepsis, we did not include sepsis developing at later points during hospitalization. We did not include organ dysfunction in the definition of sepsis. Initial review of 1,349 hospital records indicated excellent inter-rater agreement for presence of serious infection (kappa = 0.92) and the presence of sepsis (kappa = 0.90) upon hospital presentation. We assessed sepsis events occurring over a 10-year span from participant enrollment through December 31, 2012.

### Determination of Biomarker Levels

Study personnel collected blood and urine samples from all REGARDS participants at subjects’ homes following a 10–12 hour fast. Samples were centrifuged to separate serum or plasma within 2 hours of collection and shipped overnight on ice packs to the laboratories at the University of Vermont. On arrival, study personnel centrifuged the samples at 30,000 *g* and 4°C. The samples were either analyzed (general chemistries) immediately or stored at −80°C.

Serum Cyst-C and hsCRP were assessed using particle-enhanced immunonephelometry (N Latex Cystatin C, N High-sensitivity CRP, Siemens AG, Munich, Germany). In contrast with conventional CRP assays, the high-sensitivity CRP technique is able to detect levels as low as 0.04 mg/L. Serum creatinine was determined by colorimetric reflectance spectrophotometry (Ortho Vitros Clinical Chemistry System 950IRC, Johnson & Johnson Clinical Diagnostics, Raritan, New Jersey, USA). Urinary albumin was measured via nephelometry (BN ProSpec Nephelometer, Dade Behring, Siemens Healthcare, Deerfield, Illinois, USA). Urinary creatinine was measured with a rate blanked Jaffé procedure (Modular-P analyzer, Roche/Hitachi, Roche Diagnostics, Indianapolis, Indiana, USA). While not standardized to the International Federation of Clinical Chemistry and Laboratory Medicine (IFCC) standard, Cyst-C assay values were consistently 11% lower than the international benchmark.

### Participant Characteristics

REGARDS determined participant characteristics during the initial interview and in-home visit. Participant demographics included age, race, sex, income, educational attainment, and geographic location. Race was defined as white or black/African American. Income was divided into four categories (<20,000$, 20–34,000$, 35–74,000$, and ≥75,000$). Education categories included less than high school, high school graduate, some college, college or higher. Geographic region included the stroke “buckle” (coastal plains of North Carolina, South Carolina and Georgia), “stroke belt” (remainder of North Carolina, South Carolina and Georgia plus Tennessee, Mississippi, Alabama, Louisiana and Arkansas) and non-belt.

Health behaviors included tobacco and alcohol use. Smoking status included current, past and never. We defined alcohol use as moderate (1 drink per day for women or 2 drinks per day for men) and heavy alcohol use (>1 drink per day for women and >2 drinks per day for men), per the National Institute on Alcohol Abuse and Alcoholism classification [31].

Chronic medical conditions included cancer, coronary artery disease, diabetes, dyslipidemia, hypertension, myocardial infarction, obesity and stroke. Participants self-reported history of cancer, myocardial infarction or stroke. Coronary artery disease included a history of myocardial infarction or coronary intervention. Diabetes was defined as a fasting glucose ≥126 mg/L (or a glucose ≥200 mg/L for those not fasting) or the use of insulin or oral hypoglycemic agents. Dyslipidemia consisted of low-density lipoprotein cholesterol >130 mg/dL, or use of lipid lowering medications. Hypertension included a systolic blood pressure ≥140 mm Hg, diastolic blood pressure ≥90 mm Hg, or the reported use of antihypertensive agents. Obesity included a body mass index of ≥30 kg/m^2^ or waist circumference >102 cm for males or >88 cm for females.

REGARDS did not collect information on pulmonary conditions such as asthma and chronic obstructive pulmonary disease. Therefore, we defined participant use of pulmonary medications as a surrogate for chronic lung disease. Obtained from each participant’s medication inventory, pulmonary medications included beta-2 adrenergic agonists, leukotriene inhibitors, inhaled corticosteroids, combination inhalers, and other pulmonary medications such as ipratropium, cromolyn, aminophylline and theophylline.

### Data Analysis

We defined elevated Cyst-C as serum levels above the fourth quartile for the cohort (>1.12 mg/dL). We converted serum creatinine to eGFR using the CKD-EPI equation, defining eGFR levels <60 ml/min/1.73 m^2^ as abnormal [32]. We similarly determined the ACR, defining values ≥30 mg/g as abnormal. We defined hsCRP >3.0 mg/dL as elevated [33].

We compared demographic, health behavior and clinical characteristics between participants with elevated and normal Cyst-C utilizing Pearson chi-square or t-tests. We defined person-time at risk as the time (days) from first in-person examination to the first episode of sepsis, the last follow-up interview, death or December 31, 2012, whichever came first. We censored all participants who died prior to the first sepsis event.

In evaluating the independent association of elevated Cyst-C with first sepsis events, we first fit a series of Cox regression models adjusting for participant demographics, health behaviors and chronic medical conditions. Because Cyst-C has been proposed as a marker of CKD, incorporation of markers of CKD to the model would be expected to attenuate the association between Cyst-C and first sepsis event. Similarly, because Cyst-C is associated with systemic inflammation, incorporation of hsCRP to the model would be expected to also reduce the magnitude of the association between Cyst-C and sepsis. Therefore, we sequentially added abnormal eGFR and ACR, elevated hsCRP, and all three variables to the multivariable model, evaluating the changes in the hazard ratio for the association between Cyst-C and first sepsis event. Due to a substantial number of missing values for several variables (income 12.4%, Cyst-C 6.8%, hsCRP 6.4%, albumin 4.7%, creatinine 4.5%), we performed the multivariable modeling with multiple imputation using chained equations (Stata “MI” suite), pooling regression coefficient estimates for each model across 10 imputations using Rubin’s rules [34,35].

We verified the proportional hazards assumption using scaled Schoenfeld residuals as well as examining interactions with log-transformed study time. We examined multiplicative interactions between abnormal Cyst-C, eGFR, ACR and hsCRP ([Cyst-C X eGFR], [Cyst-C X ACR], [Cyst-C X hsCRP]) in the final model. We verified the absence of collinearities by examining variance inflation factors.

To test the robustness of the results, we conducted a series of sensitivity analyses. We fit a model using Cyst-C quartiles. In the primary analysis we defined eGFR, ACR and hsCRP using cutoffs commonly used in clinical practice; we repeated the analysis with eGFR, ACR and hsCRP dichotomized to their 75th percentile values. Lastly, we fit models with Cyst-C, eGFR, ACR and hsCRP as continuous variables normalized by their respective standard deviations.

We conducted all analyses using Stata 12.1 (Stata, Inc., College Station, Texas).

## Results

**Figure 1:**
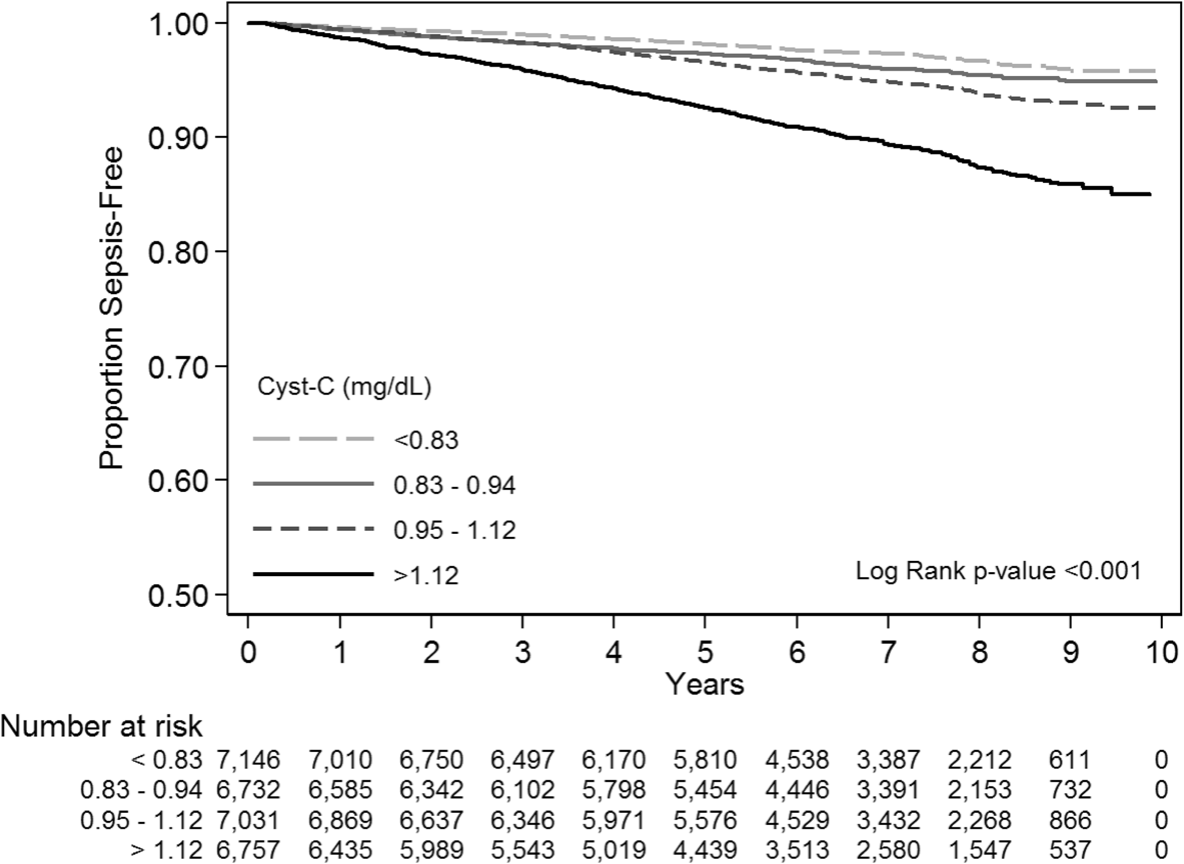
Kaplan-Meier Survival Curves for first-sepsis events, stratified by Cystatin C quartiles.

Among the 30,239 REGARDS subjects, we included 29,696 participants with available follow-up information. From February 5, 2003 through December 31, 2012 there were 3,442 hospitalizations for a serious infection, encompassing 1,856 sepsis events among 1,532 individual participants. Median follow-up time for each participant was 6.6 years (interquartile range 5.1-8.1). Sepsis-free survival was lower among those with elevated Cyst-C than those with normal Cyst-C (16.0 vs. 5.9 per 1000 person-years). (Figure 1) The most common infection types associated with the first sepsis episode were pneumonia (39.4%), kidney and urinary tract infections (17.0%), and abdominal infections (15.1 %) (Table 1).

**Table 1:** **Infection types associated with hospitalizations for sepsis**

**Infection type**	**Number of sepsis hospitalizations**
	**(n = 1,532)**
	**N (%)**
Pneumonia	603 (39.4)
Kidney and urinary tract infections	261 (17.0)
Abdominal	231 (15.1)
Bronchitis, influenza and other lung infections	138 (9.0)
Skin and soft tissue	123 (8.0)
Sepsis	104 (6.8)
Fever of unknown origin	29 (1.9)
Surgical Wound	10 (0.7)
Catheter (IV/Central/Dialysis)	6 (0.4)
Meningitis	5 (0.3)
Unknown/ther	22 (1.4)

Includes 1,532 first sepsis episodes.

Median Cyst-C levels were 1.08 (IQR 0.91-1.33) mg/dL for sepsis and 0.94 (IQR 0.82-1.10) mg/dL for non-sepsis individuals (p < 0.001); 43.8% of sepsis and 23.4% of non-sepsis individuals exhibited elevated Cyst-C >1.12 mg/dL. The Pearson correlations between Cyst-C and eGFR, ACR and hsCRP were −0.63, 0.35 and 0.15, respectively. Individuals with elevated Cyst-C were more likely to be older, male and of white race (Table 2). Individuals with elevated Cyst-C reported lower education and income levels. Elevated Cyst-C was more common among past or current smokers but less common among those with an alcohol use history. Elevated Cyst-C was more common among those with chronic medical conditions.

**Table 2:** **Characteristics of subjects by baseline Cystatin C quartiles**

**Characteristic**	**Cyst-C**	**Cyst-C**	**Cyst-C**	**Cyst-C**	p-value*
	**<0.83 mg/dL**	**0.83-0.94 mg/dL**	**0.95-1.12 mg/dL**	**>1.12 mg/dL**	
	**(n=7,146)**	**(n=6,732)**	**(n=7,031)**	**(n=6,757)**	
***DEMOGRAPHICS***					
**Age mean (SD)**	59.8 (8.0)	63.3 (8.4)	66.2 (8.8)	70.3 (9.1)	<0.001
**Sex (%)**					<0.001
Male	59.1	45.9	48.8	46.6	
Female	41.0	54.1	51.2	53.4	
**Race (%)**					<0.001
White	52.9	60.2	63.5	61.6	
Black	47.1	39.8	36.6	38.5	
**Education (%)**					
Less than high school	8.8	10.2	12.8	17.4	
High school graduate	23.6	24.8	26.3	28.7	
Some college	26.3	27.0	27.0	27.0	
College or higher	41.3	38.0	33.8	27.0	
Missing N (%)	1 (0.0)	6 (0.1)	4 (0.1)	10 (0.2)	
**Income (%)**					<0.001
<$20 k	14.8	17.2	20.9	28.1	
$20k-$34 k	22.8	25.3	28.5	33.7	
$35k-$74 k	36.3	35.3	35.0	29.7	
≥$75 k	26.2	22.2	15.6	8.5	
Missing N (%)	812 (11.4)	795 (11.8)	882 (12.5)	879 (13.0)	
**Geographic region (%)**					0.012
Non-belt/buckle	44.4	45.3	43.3	44.3	
Stroke belt	35.4	33.6	36.1	34.0	
Stroke buckle	20.2	21.1	20.6	21.8	
***HEALTH BEHAVIORS***					
**Tobacco Use (%)**					<0.001
Current	12.9	14.5	15.1	15.4	
Past	38.4	39.6	41.6	42.1	
Never	48.7	45.9	43.3	42.5	
Missing N (%)	33 (0.5)	24 (0.4)	25 (0.4)	19 (0.3)	
**Alcohol use (%)**					<0.001
Heavy	5.8	4.6	3.6	2.2	
Moderate	40.1	36.3	32.2	25.4	
None	54.1	59.1	64.2	72.5	
Missing N (%)	134 (1.9)	124 (1.8)	128 (1.8)	157 (2.3)	
***CHRONIC MEDICAL CONDITIONS***					
Cancer (%)	6.2	7.4	9.5	12.1	<0.001
Chronic lung disease (%)	7.3	8.4	9.1	11.8	<0.001
Coronary artery disease (%)	10.2	13.3	18.1	30.2	<0.001
Diabetes (%)	17.4	18.1	20.5	33.1	<0.001
Dyslipidemia (%)	49.7	56.1	61.9	67.7	<0.001
Hypertension (%)	45.8	52.7	61.5	76.8	<0.001
Myocardial infarction (%)	7.6	9.5	12.7	21.2	<0.001
Obesity (%)	43.7	50.2	56.1	62.9	<0.001
Stroke (%)	3.4	4.1	6.0	11.4	<0.001
***BIOMARKERS***					
Serum creatinine-based estimated glomerular filtration rate (eGFR) <60 mg/min/1.73m^2^ (%)	0.3	1.0	4.5	40.7	<0.001
Missing N (%)	6 (0.1)	7 (0.1)	6 (0.1)	11 (0.2)	
Urinary albumin-to-creatinine ratio (ACR) ≥30 mg/g	9.0	9.4	12.7	29.7	<0.001
Missing N (%)	193 (2.7)	202 (3.0)	220 (3.1)	333 (4.9)	
Serum high-sensitivity C-reactive protein (hsCRP) >3.0 mg/dL	31.9	36.3	42.9	51.8	<0.001
Missing N (%)	4 (0.1)	3 (0.4)	6 (0.1)	4 (0.1)	

*Based on chi-square test for categorical variables and t-test for continuous variables. Based on 29,696 participants with follow-up data available. 2,030 (6.8%) missing Cystatin C measurement.

Following multiple imputation, in unadjusted analysis, elevated Cyst-C (>1.12 mg/dL) was independently associated with increased rates of sepsis (HR 2.70; 95% CI: 2.43-3.00) (Table 3). Elevated Cyst-C remained independently associated with sepsis risk after adjustment for sociodemographics, health behaviors and chronic medical conditions (HR 1.75; 95% CI: 1.55-1.96). The association between elevated Cyst-C and sepsis persisted after adjusting for eGFR < 60 mg/min/1.73 m^2^ and ACR ≥ 30 mg/g (HR 1.57; 95% CI: 1.38-1.79). Elevated Cyst-C similarly remained independently associated with sepsis after adjustment for hsCRP >3.0 mg/dL (HR 1.67; 95% CI: 1.48-1.87). Elevated Cyst-C also remained independently associated with sepsis after adjustment for eGFR < 60 mg/min/1.73 m^2^, ACR ≥30 mg/g and hsCRP >3.0 mg/dL (HR 1.51; 95% CI: 1.32-1.72).

**Table 3:** **Hazard ratios (HRs) and 95% confidence intervals (CI) for the associations between elevated Cystatin C (Cyst-C >1.12 mg/dL) and rates of sepsis**

**Variable**	**Unadjusted Models**	Add sociodemographics*	**Add health behaviors** ^**†**^	**Add chronic medical conditions** ^**‡**^	**Add eGFR and ACR**	**Add hsCRP**	**Add eGFR, ACR and hsCRP**
Cystatin C ≤1.12 mg/dL	Referent	Referent	Referent	Referent	Referent	Referent	Referent
Cystatin C >1.12 mg/dL	2.70 (2.43-3.00)	2.12 (1.89-2.37)	2.06 (1.84-2.31)	1.75 (1.55-1.96)	1.57 (1.38-1.79)	1.67 (1.48-1.87)	1.51 (1.32-1.72)
eGFR≥60 ml/min/1.73m^2^	Referent				Referent		Referent
eGFR<60 ml/min/1.73m^2^	2.38 (2.10-2.70)	---	---	---	1.13 (0.98-1.32)	---	1.14 (0.98-1.32)
ACR <30 mg/g	Referent				Referent		Referent
ACR ≥30 mg/g	2.22 (1.97-2.49)	---	---	---	1.57 (1.38-1.79)	---	1.45 (1.28-1.65)
hsCRP ≤3.0 mg/dL	Referent					Referent	Referent
hsCRP >3.0 mg/dL	1.71 (1.54-1.90)	---	---	---	---	1.51 (1.35-1.68)	1.48 (1.32-1.65)

Estimates pooled after multiple imputation using Rubin’s rules, total of 29,696 REGARDS participants included in the analysis. *Age, sex, race, region, income, education. ^†^Tobacco and alcohol use. ^‡^History of cancer, chronic lung disease, coronary artery disease, diabetes, dyslipidemia, hypertension, myocardial infarction, obesity, stroke. eGFR = estimated glomerular filtration rate. ACR = albumin-to-creatinine ratio. hsCRP = high sensitivity C-reactive protein. Cyst-C levels ranged from 0.1 to 11.9 mg/dL.

The proportional hazards assumption was satisfied for all covariates. The multiplicative interactions between abnormal Cyst-C, eGFR, ACR and hsCRP ([Cyst-C X eGFR], [Cyst-C X ACR], [Cyst-C X hsCRP]) were not statistically significant. Based upon an examination of variance inflation factors, there was no evidence of collinearity between Cyst-C and eGFR, ACR or hsCRP.

In sensitivity analyses, we repeated the effort using quartiles of Cyst-C, the 75th percentile of eGFR (<73.1 ml/min/1.73 m^2^), ACR (≥16.1 mg/g) and hsCRP (>5.0 mg/dL), and continuous forms of Cyst-C, eGFR, ACR and hsCRP normalized by their standard deviation (Table 4). These models yielded inferences similar to the primary analysis.

**Table 4:** **Sensitivity analysis**

**Variable**	**Unadjusted**	**Model 1**	**Model 2**	**Model 3**	**Model 4**	**Model 5**
Cystatin C ≥1.12 mg/dL	2.70 (2.43-3.00)	1.51 (1.32-1.72)		1.60 (1.40-1.83)		
Cystatin C Quartiles						
Q1 (<0.83 mg/dL)	Ref		Ref		Ref	
Q2 (0.83-0.94 mg/dL)	1.40 (1.16-1.69)		1.14 (0.94-1.37)		1.14 (0.94-1.38)	
Q3 (0.95-1.12 mg/dL)	1.81 (1.51-2.17)		1.20 (0.96-1.45)		1.22 (1.01-1.47)	
Q4 (>1.12 mg/dL)	3.77 (3.20-4.45)		1.72 (1.42-2.09)		1.85 (1.51-2.27)	
Cystatin C (per SD) (mg/dL)	1.24 (1.21-1.27)					1.16 (1.11-1.21)
eGFR <60 ml/min/1.73m^2^	2.38 (2.10-2.70)	1.14 (0.98-1.32)	1.14 (0.98-1.32)			
ACR ≥30 mg/g	2.22 (1.97-2.49)	1.45 (1.28-1.65)	1.45 (1.28-1.65)			
hsCRP >3.0 mg/dL	1.71 (1.54-1.90)	1.48 (1.32-1.65)	1.47 (1.31-1.64)			
eGFR <73.1 ml/min/1.73m^2^	1.91 (1.72-2.12)			1.00 (0.87-1.14)	0.98 (0.86-1.12)	
ACR ≥16.1 mg/g	2.05 (1.85-2.28)			1.41 (1.26-1.58)	1.41 (1.26-1.58)	
hsCRP >5.0 mg/dL	1.75 (1.57-1.95)			1.51 (1.34-1.69)	1.49 (1.33-1.68)	
eGFR (per SD) (ml/min/1.73m^2^)	0.69 (0.65-0.72)					0.97 (0.90-1.04)
ACR (per SD) (mg/g)	1.07 (1.03-1.11)					1.01 (0.97-1.06)
hsCRP (per SD) (mg/dL)	1.12 (1.10-1.15)					1.10 (1.06-1.14)

Hazard ratios (HRs) and 95% confidence intervals (CI) for the associations between Cystatin-C levels and rates of sepsis.

Estimates pooled after multiple imputation using Rubin’s rules. Total of 29,696 REGARDS participants included in the analysis. Except for unadjusted model, all models adjusted for sociodemographics, health behaviors, and chronic medical conditions. eGFR = estimated glomerular filtration rate. ACR = albumin-to-creatinine ratio. hsCRP = high sensitivity C-reactive protein. Cyst-C levels ranged from 0.1 to 11.9 mg/dL.

Model 1 – Primary model; binary Cyst-C; binary eGFR, ACR and hsCRP using clinical cutpoints.

Model 2 – Cyst-C quartiles; binary eGFR, ACR and hsCRP using clinical cutpoints.

Model 3 – Binary Cyst-C; binary eGFR, ACR and hsCRP using 75th percentile as cutpoints.

Model 4 – Cyst-C quartiles; binary eGFR, ACR and hsCRP using 75th percentile as cutpoints.

Model 5 – Continuous Cyst-C, eGFR, ACR and hsCRP, normalized by standard deviation.

## Discussion

CKD and systemic inflammation are recognized risk factors for sepsis [9-11]. While Cyst-C has been studied as a marker of renal function, other studies highlight the potential role of Cyst-C as a marker of more general systemic inflammation [13,14,36,37]. In this analysis, elevated serum Cyst-C > 1.12 mg/dL measured at baseline was independently associated with future risk of sepsis events. This association persisted after adjusting for common markers of CKD (abnormal creatinine-based eGFR and elevated ACR) as well as elevated hsCRP, a marker of systemic inflammation [38]. These observations suggest that Cyst-C may indicate the presence of additional steps in the sepsis pathophysiological pathway that are not completely explained by renal disease or systemic inflammation. Our findings originate from REGARDS, one of the largest population-based cohorts in the US and encompass sepsis events over a 10-year span.

Recent attention has focused on the utility of serum Cyst-C as a marker of renal disease, with studies indicating its superiority to serum creatinine for detecting progression to end-stage renal disease and mortality [14]. However, our observations support studies suggesting that the health effects of Cyst-C are not entirely explained by renal function. For example, in a study of 8,058 persons in the Netherlands, Knight, et al. found that older age, male gender, tobacco use and higher weight, height and hsCRP were associated with Cyst-C, independent of renal function [36]. In a study of 3,418 persons, Stevens, et al. found that factors other than eGFR were associated with serum Cyst-C levels [37]. Other studies have characterized Cyst-C as a marker for inflammation in cardiovascular disease, systemic lupus erythematosus, and malignancy [15,16,18-23].

Select studies have evaluated elevated Cyst-C as a proximal risk factor for sepsis. In a study of 444 intensive care unit patients, Nejat, et al. found that elevated urinary Cyst-C was associated with five-fold increased adjusted odds of sepsis [28]. In a study of 5,142 subjects ≥65 years old in the Cardiovascular Health Study cohort, Dalrymple et al. found that reduced eGFR estimated by serum Cyst-C was associated with increased risk of hospitalization for an infection [29]. Of note, the authors found that eGFR calculated using serum creatinine did not yield the similar associations, and comment that Cyst-C may indicate additional factors beyond kidney function that are associated with infection risk. Our complementary study has important differences, including the use of a broader age range of community-dwelling adults, a longer follow-up period, Cyst-C measurements obtained at stable phase of health, and focus on hospitalizations for sepsis.

The utility of these findings is in aiding the prediction of long-term sepsis risk. Sepsis is usually conceptualized as an acute event, with current clinical and scientific efforts focused on its early detection and management [2]. However, as is the case with conditions such as myocardial infarction and stroke, prediction of sepsis risk at a stable phase of health could have enormous impacts upon its care and outcomes. For example, for individuals identified as having the highest sepsis risk, clinicians may implement more aggressive resuscitative care or have a lower threshold for intensive care unit admission. Our prior studies have identified a host of risk factors associated with increased sepsis risk including chronic kidney disease, chronic lung disease, obesity, increased high-sensitivity C-reactive protein, and even physical inactivity.[7-9,11]. While not formally evaluated in this analysis, Cyst-C could offer additional risk prediction information. Further research is needed to better define the role of Cyst-C in quantifying individuals’ long-term sepsis risk.

### Limitations

Only Cyst-C measurements taken at baseline enrollment in the REGARDS study were available for analysis. We were unable to examine Cyst-C proximal to sepsis hospitalization or repeat Cyst-C measurements. A portion of subjects did not have Cyst-C measurements; we tried to overcome this limitation through the use of multiple imputation. While Cyst-C levels were not standardized to the IFCC standard, the application of a linear adjustment would not have affected the observed inferences.

REGARDS was designed to study stroke, not sepsis. REGARDS is not a surveillance study, and thus we may not have identified all sepsis events. However, misclassification of sepsis events should be similar between Cyst-C levels, and thus the analysis should present underestimates of the true association. Early death may act as a competing risk, preventing an individual from experiencing sepsis events. Those with elevated Cyst-C exhibited higher rates of death than those with normal Cyst-C; thus, we would expect an even stronger association with sepsis risk if we were to fully account for the competing risk of death.

By design, the REGARDS cohort includes only African Americans and whites, and thus these results may not generalize to other ethnic groups. We examined the presence of comorbid conditions but not their severity. The REGARDS cohort contains individuals over 45 years old only; sepsis rates and the observed associations may have differed for younger individuals. We identified individuals who presented to the hospital with sepsis, but did not include those who acquired sepsis during their hospitalization.

While we adjusted for a range of potential confounders, the observed associations may have been influenced by other variables such as vaccination or access to healthcare. As with all observational studies, residual confounding is always a concern. However, our risk adjustment strategy accounted for a comprehensive range of variables that were systematically identified for each participant at the beginning of the REGARDS study. We could not account for changes in these patterns over time.

## Conclusion

In this study elevated serum Cyst-C measured at baseline was independently associated with future risk of sepsis. This association persisted event after adjustment for markers of kidney disease and systemic inflammation. Cyst-C may play a potential role in sepsis risk prediction and reduction.

## Abbreviations

CKD: Chronic kidney disease
Cyst-C: Cystatin C
eGFR: Estimated glomerular filtration rate
ACR: Albumin-to-creatinine ratio
hsCRP: High-sensitivity C-reactive protein
REGARDS: REasons for Geographic and Racial Differences in Stroke
HR: Hazard ratio
CI: Confidence interval
SIRS: Systemic inflammatory response syndrome
PCO2: Carbon dioxide partial pressure
